# Targeting Macromolecules to CNS and Other Hard-to-Treat Organs Using Lectin-Mediated Delivery

**DOI:** 10.3390/ijms21030971

**Published:** 2020-02-01

**Authors:** Walter Acosta, Carole L. Cramer

**Affiliations:** BioStrategies LC, PO Box 2428, State University, Jonesboro, AR 72467, USA; cramer@biostrategies-lc.com

**Keywords:** enzyme replacement therapy (ERT), BBB, RTB, lectin, macromolecule transport, lysosomal storage diseases, CNS

## Abstract

The greatest challenges for therapeutic efficacy of many macromolecular drugs that act on intracellular are delivery to key organs and tissues and delivery into cells and subcellular compartments. Transport of drugs into critical cells associated with disease, including those in organs protected by restrictive biological barriers such as central nervous system (CNS), bone, and eye remains a significant hurdle to drug efficacy and impacts commercial risk and incentives for drug development for many diseases. These limitations expose a significant need for the development of novel strategies for macromolecule delivery. RTB lectin is the non-toxic carbohydrate-binding subunit B of ricin toxin with high affinity for galactose/galactosamine-containing glycolipids and glycoproteins common on human cell surfaces. RTB mediates endocytic uptake into mammalian cells by multiple routes exploiting both adsorptive-mediated and receptor-mediated mechanisms. In vivo biodistribution studies in lysosomal storage disease models provide evidence for the theory that the RTB-lectin transports corrective doses of enzymes across the blood–brain barrier to treat CNS pathologies. These results encompass significant implications for protein-based therapeutic approaches to address lysosomal and other diseases having strong CNS involvement.

## 1. Introduction

Biologics represent the fastest-growing sector for new drug development. Most of these drugs, for example immuno-therapeutics, typically act on targets in the blood or localized at the cell surface. However, mobilizing large therapeutic molecules into and across endothelial/epithelial cell layers to reach complex intracellular targets present in distal cells remains a challenge. Delivering these products across the blood–brain barrier (BBB) to treat pathologies of the central nervous system is particularly problematic and new strategies are clearly required to address diseases having severe neurological manifestations. Lectins such as the plant lectin RTB show significant promise as protein carriers with the potential to address delivery to CNS as well as delivery to other hard-to-treat tissues and cells. The term lectin refers to a group of proteins that binds to specific sugars. The surface of human cells is covered with complex glycolipids and glycoproteins providing abundant targets for lectin binding. Following binding, lectins such as RTB direct efficient endocytosis and transcytosis, delivering associated cargo to lysosomes or across cells for delivery to adjacent cells. The unique mechanistic attributes of lectin-based carriers differ significantly from those of current protein delivery approaches, which typically depend on specific receptor interactions to direct uptake into cells and lysosomes. RTB’s ability to access a broad array of cell types, to traverse multiple cell layers, and to place cargo enzymes in lysosomal compartments make it particularly well suited for enzyme-based treatments of lysosomal diseases. This review highlights current unmet needs in treating the neurodegeneration and other CNS manifestations prevalent in lysosomal diseases, the promise of the RTB lectin to support a new class of delivery-enhanced enzyme replacement therapies (ERTs) based on human enzyme:RTB fusions, and the broader implications of this emerging technology for delivery of biologic drugs.

## 2. Treating the CNS–A Significant Unmet Medical Need

The demand for more effective drug delivery to the central nervous system remains high. Neurological disorders such as Alzheimer’s, Parkinson’s, and multiple sclerosis, among others, constitute 16.8% of total deaths globally [1]. Many promising drugs with the potential to treat CNS disorders fail to enter the clinical development phase due to failure to deliver adequate quantities of the active molecule to the CNS, thus leaving many diseases undertreated. Many rare genetic disorders also manifest with significant progressive neurodegeneration and have served as models for the development of novel strategies to address trans-BBB drug delivery and treatment of CNS pathologies.

Effectiveness of therapeutic options depends on the drug’s bioactivity, but equally important is its ability to reach critical targets within tissues and cells linked with pathology. Intravenous administration is the simplest and least invasive method for systemic delivery of drugs by infusing molecules directly into the circulatory system. The blood supplies all cells and tissues with vital elements and nutrients. However, the delivery of macromolecules such as proteins from circulation into adjacent cell layers is limited by a variety of biological barriers and selectivity of cell uptake mechanisms. Macromolecular transport across plasma membranes and intracellular membranes is regulated by integral transmembrane channels or carriers. For any particular drug, the specific components required for uptake might be limited or lacking, especially in specialized tissues such as the endothelium of the central nervous system.

**Figure 1:**
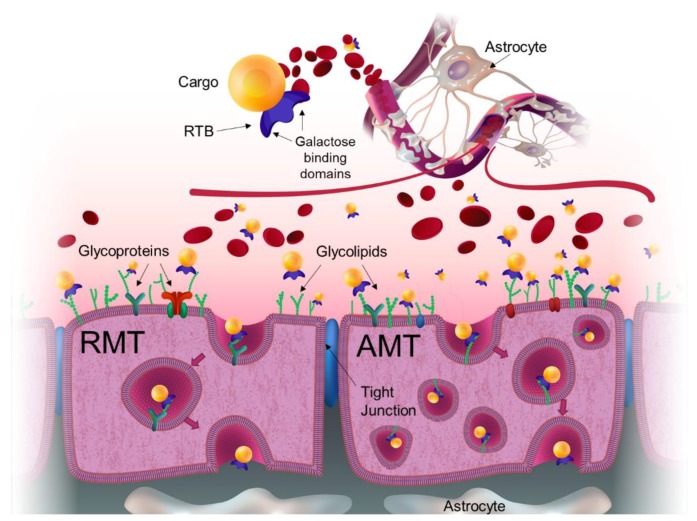
Pathways utilized by RTB lectin across the blood–brain barrier. Blood-borne lectin:cargo fusion product reaches the cerebral endothelial membrane or blood–brain barrier (BBB) when administered intravenously. BBB is a capillary endothelial layer formed by specialized cells with highly selective expression of receptors and transporters designed to maintain the integrity of the chemical environment within the brain. These cells are glued together with tight junctions that restrict the penetration of water-soluble compounds. RTB lectin binds to any glycoprotein or glycolipids with exposed galactose residues, which are highly abundant on the apical surface of cell membranes, triggering endocytosis and transcytosis. This typically takes the form of adsorptive-mediated transcytosis (AMT). However, because RTB also binds to the glycan component of an existing surface receptor, RTB can exploit receptor-mediated endocytosis/transcytosis (RMT) mechanisms to deliver cargo across the BBB.

Neuronal signaling within CNS requires a highly controlled chemical environment. Multiple protective mechanisms have evolved to regulate access into the brain. Exogenous molecules interface with at least three distinct barriers that modulate transport into the brain: the blood–brain barrier (BBB), the blood-cerebrospinal fluid (CSF) barrier, and the arachnoid barrier [2]. The BBB is created by the vascular endothelial cells, which form a relatively impenetrable wall of cells cemented by tight junctions (Figure 1). This network of specialized capillaries comprises the largest interface for blood–brain exchange with a total surface area of 12–18 m^2^ in humans [3]. Transport through this wall is highly controlled and most molecules lack the required chemical characteristics to gain access to the brain. Molecules in the blood gain access to the brain by exploiting selective cell surface transporters or by free diffusion if the molecule is lipophilic and has a molecular weight lower than 400 Da [4]. Most macromolecules, including proteins and infectious agents such as viruses, are physically prevented from entering the brain by the tight junctions and the limited presence of selective receptors within this specialized barrier. 

Recognizing the challenges of treating the CNS, various strategies to deliver therapeutics to the brain include neurosurgical-based interventions which encompass invasive transcranial procedures; chemical-based strategies which consist of increasing the lipid solubility of the molecule; and biology-based strategies which require drug modifications to use vesicular mechanism regulated by endogenous BBB transporters [4]. These vesicular mechanisms involve either receptor-mediated transcytosis (RMT) or adsorptive-mediated transcytosis (AMT) [2]. In addition to paucity and limited selectivity of receptor targets on cells comprising the BBB, there is also reduced vesicular trafficking within these cells. Thus, directing efficient directional transcytosis across the BBB to provide therapeutic protein presentation to neurons and glial cells of the CNS remains problematic.

## 3. Lysosomal Storage Diseases and the Need for Widespread Biodistribution of Therapeutics

Lysosomal storage disorders (LSD) are autosomal recessive inherited pathologies caused by mutations in genes that encode proteins that are essential for macromolecule degradation in lysosomes. Around 83 lysosomal storage disorders have been described that encompass about 50 different enzyme deficiencies [5]. The lack of any one of these enzymes can cause chaos in the lysosomal system, including substrate buildup, aberrant intra-lysosomal protein accumulation, changes in lysosome number, lysosomal membrane permeabilization, accumulation of undegraded autophagosomes, aberrant exocytosis, and translocation of soluble lysosomal components into the cytosol [6]. These cellular changes lead to significant pathologies and disease manifestations. Since these genetic mutations affect all cells in the body, pathologies are typically progressive and show multisystem involvement. However, for many of these diseases, pathological manifestations are more pronounced in specific organs or tissues depending on the sites where the target substrate is highly synthesized or accumulated. Although LSDs are individually rare, collectively they affect 1 in 5000 live births [5] and have significant impacts on patients, their families, and national health care costs.

Enzyme replacement therapies (ERT) involve providing the patient with an infusion containing the corrective enzyme, typically at 1- to 2-week intervals. ERTs are the treatment of choice for soluble hydrolase deficiencies and at least 10 LSDs currently have approved ERTs [7]. Small-molecule therapies, including substrate reduction and chaperone therapies are also approved for some LSDs. Gene therapy and genome editing treatments have recently entered clinical trials for several lysosomal diseases [5]. 

Currently approved ERTs are manufactured as recombinant glycoproteins expressed in transfected Chinese hamster ovary (CHO) cells, human fibroblasts, suspension-cultured plant cells, or egg whites from genetically engineered hens, with the large majority being mammalian cell-derived products. Cell uptake and lysosomal delivery of all these ERTs are orchestrated by binding to cell surface receptors, either the mannose-6-phosphate receptors (M6PRs) or the high mannose receptor (MMR) via M6P-terminated or mannose-terminated glycans present on the recombinant protein. With the exception of ERTs for Gaucher Disease, all currently approved ERTs use M6PR receptors for lysosomal delivery. The primary cellular role of M6PRs is to capture lysosomal enzymes within the Golgi and ensure trafficking to pre-lysosomal endosomes to protect other compartments from their degradative activities. Thus, M6PRs are found in the trans-Golgi network (TGN) and endosomes, with a small amount (3–10%) localized at the plasma membrane to recover misdirected hydrolases [8]. There are two types of M6PR: the cation-dependent CD-M6PR with a molecular mass of 46 kDa and the cation-independent CI-M6PR with a molecular mass of 300kDa. They both play an important role not only in sorting of lysosomal hydrolases but also in cell growth regulation, motility and other important biological functions such as tumor suppression. Due to slightly alkaline extracellular environment (pH 7.4), only CI-M6PR is capable of binding exogenous M6P-containing ligands and supporting endocytosis [9]. CI-M6PR that has localized to the cell surface is, therefore, the receptor target exploited by M6P-based ERT drugs to direct exogenous enzymes into cells and lysosomes. Other enzymes and proteins can be sorted to lysosomes by M6P-independent receptors such as the lysosomal integral protein LIMP-2, sortilin, and megalin [8,10]. It is possible that additional receptors might exist and be involved in lysosomal sorting [8] which might expand the options for delivery strategies. 

Like any other drug, efficacy of enzyme replacement strategies depends upon the delivery of the therapeutic enzyme to the appropriate tissues and cells where the substrate accumulates to pathogenic levels. Whereas ERTs have been very beneficial for patient care and quality of life, experience with current ERT drugs has identified several organs, tissues and cell types that do not receive sufficient ERT doses to correct or delay disease progression. These sites have been collectively termed “hard-to-treat” tissues that include cells within brain and nervous system; joints, bone, cartilage, and myocytes of the musculoskeletal system; and organs such as the eye. Delivery to these sites is challenging due to presence of biological barriers that support them and the limited accessibility or absence of target receptors in the endothelium of these organs. Out of all LSDs described up to date, the majority display pathological manifestations in these hard-to-treat organs and treatment options using intravenous administration of the enzyme has shown very limited success or failed to prevent these devastating aspects of disease progression [11,12,13]. Even specialized cells within organs where the enzyme is distributed can remain untreated, as is often the case for heart cardiomyocytes and podocytes of the kidney [14]. Thus, DELIVERY and the ability to traverse specific biological barriers is paramount in designing effective disease treatments. This is critical for many biologics but is also important for newly emerging technologies such as gene therapy, where the vectoring capsids fail to obtain a broad distribution within the body and within key organs and cells. 

Treatment of LSDs by replacing or expressing functional versions of the enzyme encounters another important issue: the development of antibodies against the newly presented protein. These anti-drug antibody responses (ADA) hinder treatment efficacy of the majority of ERTs available to patients by altering catalytic activity, cell uptake, and biodistribution [15,16,17]. Over 90% of the patients with Hurler (MPS I), MPS VI, Pompe and Fabry develop antibodies to their ERT drug [16]. Infants and children are most affected, as patients with early onset forms typically have no residual enzyme (i.e., are cross-reactive immunological material negative; CRIM-) and quickly develop neutralizing antibodies that abrogate therapeutic correction and can precipitate severe adverse clinical effects. Impacts on Pompe patients are often cited as the most dramatic example, where children show significant improvement in motor milestones in response to ERT only to have these effects rapidly reversed upon induction of neutralizing antibodies, leading eventually to patient death [16,18,19]. Tolerization protocols have been tested for patients where ERT efficacy is blocked by immune responses but these treatments might not be efficacious [20], are intensive for patients, and pose significant risks of infection or malignancy [16]. Gene therapy approaches are currently under development for LSDs as an alternative to ERTs. However, therapeutic enzymes produced via gene therapy also lead to ADA responses in CRIM- patients and animal models of the diseases [21], underscoring the need to develop new approaches to address challenges imposed by immune responses to therapeutic proteins. 

## 4. Lectin-Mediated Delivery: RTB as a Protein Carrier

Lectins are a very diverse group of proteins that reversibly bind carbohydrates with high specificity to the chemical structure of the glycan but have no catalytic activity [22]. Lectins are proteins expressed in multiple organisms including virus, bacteria, algae, fungi, plants, and animals. They play many different biological roles including defense, signaling, agglutination, and transport.

Most cell surface proteins and many lipids in cell membranes are glycosylated. These glycans are binding sites for lectins and depending on the lectin structure or sugar-specificity, this interaction can direct transport to the cytosol, organelles or transcytosis, typically by vesicular trafficking [23]. Affinity of the proteins for cell surface glycans has been studied for many years to exploit drug delivery to the gastrointestinal track, mucosal surfaces, lungs, eyes, and brain [23,24]. A recent example of this is the delivery of the AAV9 vector across the blood–brain barrier, which take place by interactions of the viral capsid with galactose residues on the endothelium surface [25,26]. 

Delivery of macromolecules to the brain using lectin wheat germ agglutinin (WGA) was reported by Broadwell in 1989. WGA conjugated with horseradish peroxidase (HRP) was used as an enzymatic tracer within tissues. WGA binds to cell surface sialic acid and N-acetyl-glucosamine for entry to cells by adsorptive-mediated endocytosis. In this work, intravenously administered WGA-HRP in rats and mice was detected in the parenchyma of the neurohypophysis and that of the anterior pituitary lobe after one hour. At 24 h, the protein reached the dura matter. Pericytes were labeled with WGA-HRP between 6 and 24 h after the injection. These results provided the first direct evidence of delivery of blood-borne molecules through the BBB into the brain using lectin-mediated uptake. Plant lectin RTB, naturally present in castor bean seeds (*Ricinus communis*), mediates endocytic uptake into mammalian cells, transcytosis, and trafficking to lysosomes or endoplasmic reticulum (ER) of associated proteins. RTB is the non-toxic domain of the ricin toxin. Ricin is a Type II ribosome-inactivating protein that contains two structures. One sub-unit (RTA) is a ribosome-inactivating protein known to be cytotoxic. In contrast, the RTB component mediates binding to the target cell surfaces and promotes uptake but has no intrinsic toxicity or catalytic function [27]. When ricin is synthetized by plant cells, the molecule is directed to the vacuole where it gets processed into two sub-units that are linked by a disulfide bond. Under reducing conditions, these sub-units can be separated, and both retain their bioactivity. However, in the absence of RTB, the cytotoxicity of RTA is significantly reduced due to its inability to reach the intracellular space [28,29]. Ricin has been widely studied for decades to understand its cellular sorting, physiological impacts, and strategies to develop antidotes [30]. It served as a valuable tool for the discovery and delineation of selective endocytic routes, mechanisms, and subsequent vesicular trafficking within mammalian cells [31,32]. These efforts have provided important information regarding its uptake, immunogenicity, and trafficking within mammalian cells, serving as the foundation for the development of RTB as a unique and promising delivery platform technology [32]. 

RTB is a galactose-specific lectin comprised of 262 amino acids with an approximate size of 34 kDa. The crystal structure of ricin has been determined (PDB entry 2AAI) [33] and indicates that RTB contains two lobes, each of which binds sugars. Both sites bind to galactose in an independent manner and both must be simultaneously modified to abolish lectin activity. The 2γ domain is demonstrated to also bind to N-acetylgalactosamines [34]. A single human cell contains millions of galactose binding sites on its surfaces [35,36,37] and this promiscuous binding to many different cell surface components enables exploitation of multiple intracellular trafficking pathways following endocytosis [28]. 

The RTB lectin has been shown to use all of the endocytotic routes described to date and to enter and carry associated cargo into all cell types tested [32,38,39,40,41]. RTB enters cell partially from coated pits, but mostly is internalized by clathrin-independent endocytosis (CIE) [37,42], which is responsible for approximately 70% of fluid uptake into cells [43]. In contrast to coated pits, CIE provides a high degree of versatility and speed by coordinating membrane retrieval with exocytosis rates [44]. Some of them can use dynamin (e.g., caveolae; RhoA/Rac1; toxin-induced tubules with endophilin A2; fast endophilin A2-dependent endocytosis (FEME) or use small GTPases instead of dynamin for the vesicle scission at the plasma membrane (Cdc42/Graf1; CtBP/BARS, micropinocytosis) [31,44]. For example, ricin (RTB delivered RTA) is equally efficiently internalized in both dynamin-dependent and dynamin-independent endocytic pathways, although the majority traffics by dynamin-independent routes to reach the lysosomes [45]. 

Upon endocytosis, RTB shuffles to early endosomes where the majority traffics to lysosomes or can be transported back to the cell surface facilitating transcytosis and translocation across cell layers [37,41,46]. Transcytosis occurs when the proteins avoid the lysosomal degradation pathway presumably due to the type of glycosylated targets and endocytic route of the vesicles to which the lectin has bound. This transcytotic route is fundamental for the penetration of cargo through tissues. The ability of RTB to deliver across mucosal surfaces is well established, with evidence of toxin delivery via ingestion, inhalation, nasal and subcutaneous routes leading to delivery into the circulatory system for broad distribution [47,48]. The transmucosal transport of RTB has been explored for protective antigen delivery in oral vaccine development [23,49,50]. What is less well described is the ability of RTB to transport cargo across the BBB and other protective barriers that define the “hard-to-treat” organs, tissues, and cells. Initial support of RTB’s ability to access these tissues is evidence of ricin penetration in brain and myocardium [48,51]. 

Hard-to treat organs, such as brain, need to obtain a significant number of macromolecules that they cannot synthesize for themselves but must import from the vascular system. This requires specific mechanisms to cross the various protective barriers. The endothelial cells that make up the BBB have a paucity of receptors (e.g., very low MMR and M6PR levels) and significantly reduced vesicular trafficking. The transport of transferrin, insulin, and other critical macromolecules is facilitated by the expression of selective receptors for these molecules in the apical surface of membranes. In efforts to exploit these receptors for ERTs, lysosomal enzymes have been linked to antibodies or antibody fragments that target the transferrin or insulin receptors for trans-BBB delivery to address CNS pathologies. Use of the antibodies to the human insulin receptor, known as the “Trojan Horse” strategy, has shown success in delivering enzyme and other large cargos across the BBB including delivering iduronidase to the brains of MPS I mice and primates [52,53]. This approach exploits receptor-mediated endocytosis and transcytosis mechanism (RMT), which are dependent upon receptor abundance, receptor cycling efficiency, and intracellular vesicular trafficking route into and across the BBB as discussed above. Lectin-mediated transcytosis provides distinct advantages for trans-BBB delivery supporting non-specific binding, affinity for many targets (even on brain capillary endothelial cells) and consequent low uptake saturability. The RTB lectin-delivery module is not limited by binding to a specific receptor. Instead, it binds to any glycoprotein (including receptors) or glycolipid having exposed galactose/galactosamine, triggering endocytosis/transcytosis by either adsorptive-mediated (AMT) or receptor-mediated (RMT) processes (see Figure 1). 

The ability of RTB to internalize large molecules several times its size is remarkable. When streptavidin-coupled Quantum dots were coupled to biotinylated RTB (RTB:QGs hydrodynamic diam = 30nm), they were endocytosed by dynamin-dependent and macropinocytosis-like pathways [54]. RTB even directs endocytosis into cells when its cargo enzyme is bound with enzyme-specific polyclonal antibodies, each having a mass five times greater than RTB (further described below) [55,56,57]. Thus, the RTB lectin offers numerous mechanistic features, summarized in Table 1, that may be highly beneficial for delivery of therapeutic cargo such as replacement enzyme for lysosomal storage diseases. 

## 5. RTB for Delivery of Lysosomal Enzymes

To determine whether RTB can direct broad systemic distribution including delivery to the CNS, the RTB lectin has been genetically fused to several human lysosomal enzymes, essentially replacing the RTA subunit with a specific enzyme at the N-terminal region of the lectin (enzyme:RTB). Enzymes for three diseases in which the enzymatic deficiency leads to progressive and potentially severe brain pathologies have been studied. These enzymes include α-L-iduronidase (IDUA:RTB) for MPS I - Hurler, N-sulfoglucosamine sulfohydrolase (SGSH:RTB) for MPS IIIA, and β-galactosidase (β-gal:RTB) for GM1 gangliosidosis. The RTB:enzyme fusions were synthesized in a plant-based bioproduction system [58,59,60], which supports N-linked glycosylation for both the RTB carrier (2 sites) and its human glycoprotein cargo. While plants synthesize both complex and mannose-terminated glycans, they do not produce M6P-glycans. Biochemical analyses demonstrated that the fusion proteins retained both RTB-lectin binding specificity and the respective lysosomal enzyme activity. In vitro uptake studies using human patient fibroblasts confirmed efficient cell uptake and lysosomal delivery by a mechanism independent of MMR and M6PR. However, adding lactose to the culture media inhibited the uptake of fusion protein by 99%, confirming that the RTB’s lectin activity is the main uptake driver [58,59,60]. Uptake kinetics of recombinant enzymes can be measured on cultured cells by measuring intracellular activity after incubation with increasing concentrations of protein. Maximal uptake capacity, calculated using Michaelis-Menten analysis (V_max_), is defined as the maximal intracellular activity achieved after saturation of the system. As expected based on mechanisms of adsorptive-mediated endocytosis, the enzyme:RTB products showed much lower uptake saturability and higher uptake capacity compared to the analogous mammalian-cell-derived human enzymes that depend on the M6P receptor to mediate endocytosis (e.g 20-fold higher IDUA intracellular activity on IDUA^-/-^ fibroblast) [58,59,60]. Upon delivery of the enzyme:RTB product to lysosomes, the RTB lectin component is rapidly cleaved and degraded; the respective enzyme precursors are processed to their typical mature forms. The efficient removal of RTB in the lysosome presumably minimizes the potential of the “carrier” to interfere with protein-protein and protein-substrate interactions important for processing, stability, and function of the lysosomal hydrolase. The removal of RTB and correct processing of the cargo enzyme have been seen in either N- or C-terminal lectin fusions, generating equivalent intracellular activity from the mature enzyme [60]. 

Because RTB exploits multiple endocytic mechanisms (see above), it participates in a diversity of intracellular vesicular trafficking pathways supporting directional transcytosis as well as lysosomal delivery. Transcytosis efficiencies of β-Gal:RTB have been compared to M6PR-delivered β-Gal in vitro using multi-cell layer transwell assays comprising a tight polarized β-gal^-/-^ human endothelial cell layer in the transwell insert and β-gal^-/-^ patient fibroblast cells as the recipient cell layer. Up to 60% of the β-Gal:RTB added to the apical surface was transported across the endothelial layer based on β-Gal activity and GM1 ganglioside reduction in the distal fibroblast cells, with RTB delivering 4–20 times more β-galactosidase enzyme activity compared to M6PR-delivered enzyme [61]. Overall, these in vitro studies support the hypothesis that the RTB delivery module will provide unique and advantageous pharmacological characteristics compared to ERTs currently approved (see Table 1) or in development that are dependent on (and limited by) receptor-mediated mechanisms.

Preclinical animal studies of enzyme:RTB fusions using the correspondent enzyme-deficient mouse models have provided evidence of broad systemic biodistribution of the cargo molecules to all organs tested including the central nervous system. The first indication of lysosomal enzyme delivery to the CNS by RTB lectin was evidenced in a collaboration of BioStrategies LC with Dr. Alessandra d’Azzo of St. Jude Children’s Research Hospital in the GM1 gangliosidosis mouse model. After intravenous administration of 150ug/mouse (6–7.5 mg/kg) β-Gal:RTB fusion, β-galactosidase enzyme activity was detected in the CNS and mature forms of β-galactosidase were immunodetected in homogenates of cerebellum and spinal cord demonstrating trans-BBB delivery of the enzyme and correct processing within the lysosomes of the brain cells [62]. Significant increase in enzyme activity has been also demonstrated in cerebrum, cerebellum and spinal cord of SGSH^-/-^ (MPS IIIA) and IDUA^-/-^ (MPS I) mouse models after injection of the corrective enzyme fused to RTB lectin at 12 mg/kg and 2mg/kg respectively [63,64]. Brain disease correction has been demonstrated by long-term administration of the fusion product. In a trial subcontracted to the University of Minnesota by BioStrategies, MPS I mice, treated weekly with iduronidase fused to RTB (IDUA:RTB) for 8 weeks, showed normalization of substrate levels in the brain. Treatment of CNS pathologies was further demonstrated by correction of learning and memory deficits of the mouse model [63,65]. Among these three disease models, mouse studies have consistently shown elevated enzyme activity, evidence of lysosomal delivery and enzyme distribution in all tissues tested including CNS (manuscripts in preparation).

**Figure 2:**
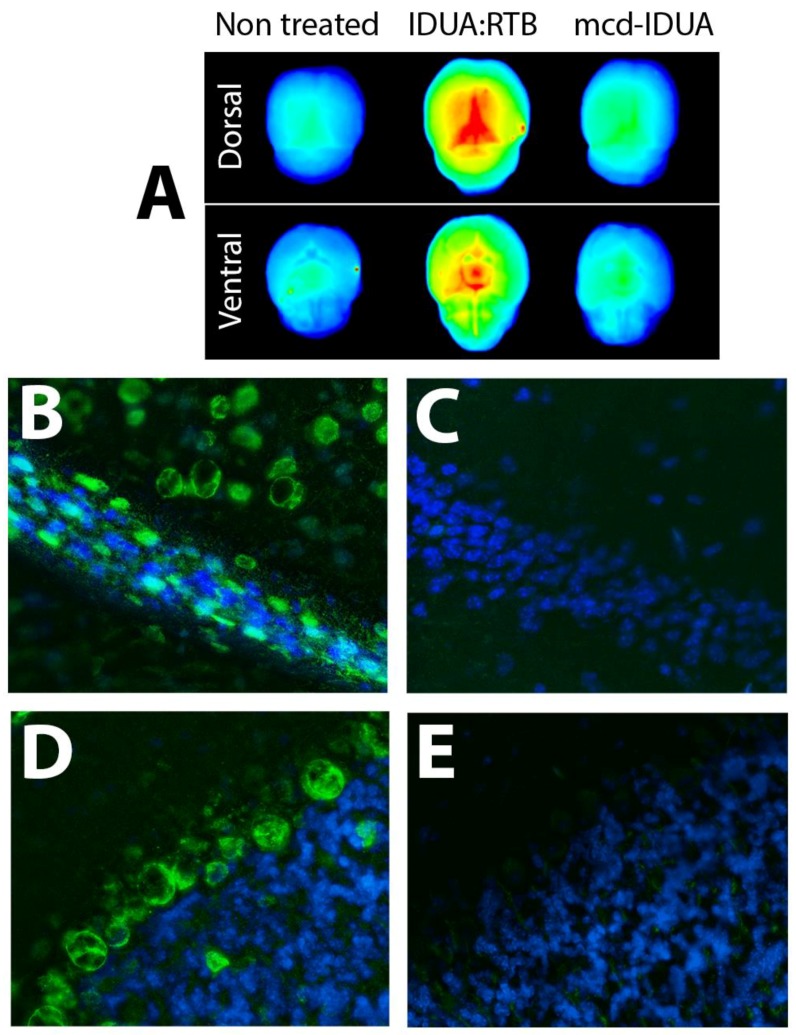
Delivery of IDUA:RTB to CNS. (**A**) Comparative imaging of brains following treatment with labeled product. Brains of mice harvested 24 h after intravenous administration of 2mg/kg of IRDye^®^ 800CW-labelled IDUA:RTB or mammalian-cell-derived IDUA (mcd-IDUA, R&D Systems) were analyzed by infrared fluorescence (800nm) imaging. A non-treated mouse was processed in parallel as control. (**B**–**E**). Immunostaining of brain using anti-RTB antibodies. Brain slices from IDUA^-/-^ mice treated intravenously with 2mg/kg of IDUA:RTB or PBS were stained with anti-RTB antibodies (Green) and counterstained with DAPI (Blue). Images were acquired and processed using identical settings. RTB protein was detected in hippocampus (**B**) and cerebellum (**D**) of treated animals. Sections from PBS treated animals were carried in parallel as controls (**C**,**E**).

Direct demonstration of lectin-mediated delivery of IDUA to the brain has recently been verified by fluorescence imaging studies (Figure 2; unpublished results). In order to compare RTB-mediated versus M6P-mediated delivery of IDUA, eight weeks old heterozygous IDUA^+/-^ mice were intravenously injected with 2mg/kg of fluorescently labelled (IRDye^®^ 800CW) IDUA:RTB or mammalian-cell-derived IDUA (mcd-IDUA, R&D Systems). An age-matched untreated animal was used as a negative control. Mice were analyzed at 24 h, a time previously demonstrated to have no residual enzyme activity in serum after IDUA:RTB infusion at this dose, [65]. At harvest, animals were perfused with saline, and the three cerebrums were imaged together for fluorescence in the near-infrared (NIR) spectrum using a Li-Cor^®^ imager. As shown in Figure 2A, dorsal and ventral images of the brains from mice treated with IDUA:RTB show a significant increase in the florescence compared to brains from non-treated animals and animals treated with fluorescently labeled mcd-IDUA. While midbrain and interbrain localization may account for most of the fluorescence intensity, increased IDUA:RTB product accumulation is evidenced throughout comparative regions of the brain including cerebral cortex. In a second experiment, eight weeks old IDUA^-/-^ mice were treated intravenously with 2mg/kg of IDUA:RTB or phosphate buffer saline (PBS; negative control) and imaged by fluorescence-based immuno-histochemistry. Four hours post-injection the mice were perfused with PBS, and the brains harvested. Cryo-sliced brain sections were immuno-stained with rabbit polyclonal anti-RTB antibodies and anti-rabbit CY7 antibodies. Hippocampus and cerebellum regions were selected for imaging as shown in Figure 2B,D. RTB protein staining was associated with multiple cell types supporting trans-BBB delivery and efficient penetration of the molecule into different regions within brain. Image statistics analysis demonstrated significant differences in the means of intensity values of RTB staining in hippocampus (B:2835 ± 1.15 C:1283 ± 0.19) and cerebellum (D:2183 ± 1.22 E:1481 ± 1.53) for both regions; *p* < 0.0001 by *t*-test. These studies validate the delivery of therapeutics to the CNS and demonstrate broad distribution of the enzyme across different cell types within the brain tissue. They also corroborate the therapeutic outcomes of the long-term treatment in the MPS I mouse model that demonstrated reduction of disease substrate within the CNS and normalization of learning and memory impacts [65].

## 6. Immunogenicity of RTB-Mediated Treatment

Many patients, especially CRIM- individuals, develop anti-drug antibodies to their ERT drugs that can alter uptake and biodistribution. Thus it is of interest to assess the immunogenicity of the enzyme:RTB fusions in treated animals. In long-term administration trials with RTB-enzyme fusions, anti-RTB and anti-cargo enzyme immunoglobulin levels in terminal serum have been measured by ELISA. Consistent with data for infusions with recombinant mammalian-cell-derived enzyme [66,67,68], elevated levels of total IgGs reacting against the cargo were detected in the respective KO mice treated with enzyme:RTB. In contrast, ELISA using recombinant RTB as a capture molecule shows no antibody response against the RTB lectin carrier [63,69]. This lack of immunogenicity of RTB is intriguing and has significant implications for lectin platform technology [70]. Low immunogenicity to RTB (as opposed to RTA) has been noted previously in ongoing efforts by public health and biodefense investigators to develop a protective vaccine against ricin toxin [71,72]. Efforts to use RTB alone as a protective antigen have been unsuccessful and it has been reported that out of the few identified RTB antibodies, only a very small proportion are capable of neutralizing the toxin uptake [57]. The two carbohydrate recognition domains of RTB are separated by approximately 75Å [73], making it difficult for a single antibody to occlude both domains simultaneously [55]. Rapid RTB degradation within lysosomes may also contribute to low immunogenicity [59,63,70]. Of significance for ERT strategies, the presence of ADA directed against the cargo protein did not block RTB-mediated uptake of the fusion protein (in contrast to mcd-enzyme) into patient fibroblasts or in enzyme-immunized KO mice [64]. Analogous corrective doses of the enzyme were delivered by RTB to all organs tested, including the CNS, in enzyme-immunized KO mice [64,70]. These promising preliminary data suggest that RTB may mitigate impacts of ADA in chronically treated patients and maintain critical biodistribution and treatment efficacy of its enzyme cargo with long-term use.

## 7. Summary

The RTB lectin displays significant promise as a carrier module for enzyme replacement therapeutics and other macromolecular drugs requiring broad biodistribution throughout the body, intracellular delivery, transport across multiple cell layers, and access to hard-to-treat tissues such as the brain. In addition to its potential to treat the key cells of the CNS and musculoskeletal systems, RTB has other beneficial features including a large payload capacity, distinct receptor-independent tissue biodistribution, rapid degradation upon lysosomal delivery, and low immunogenicity. Considering the overall pharmaceutical market, small molecules have been traditionally used for drug development. Research and development efforts have not traditionally favored proteins as therapeutics candidates due to delivery constraints, especially in pathologies with orthopedic, cardiovascular, neurological, and ocular manifestations. Lectin-mediated delivery technology could provide a novel platform for the development of treatments using molecules that had thus far been considered improbable for therapy. LSD therapies encounter different obstacles due to a variety of diseases with different organ system involvement [74] and current treatment options suffer from this pathological heterogeneity. The majority of LSD remains without an effective therapy, particularly those with CNS involvement [5]. Thus, the societal burden of these and thousands of other rare diseases that have no present treatment options could potentially be benefited by this platform technology. Scientifically, this lectin-based macromolecule delivery system also opens a whole new avenue of possibilities for research in studying the function of the brain and central nervous system. In the future, a diverse array of experimental proteins, nucleic acids, and other large molecules could be delivered to the brain as therapeutics or as probes in studies directed towards understanding how the brain functions.

## Figures and Tables

**Table 1:** Uptake and trafficking characteristics of lysosomal enzymes as directed by the RTB lectin carrier versus M6P-based mechanisms (as referenced in text).

Feature	RTB-Mediated Delivery	M6P-Mediated Delivery
**Cell Uptake**		
**Surface binding target**	Glycoproteins/glycolipids	Cation-independent M6P receptor (M6PR)
**Target distribution**	Common on all cell types	Restricted to cells having surface M6PR
**Uptake mechanisms**	Adsorption- and receptor-mediated endocytosis	Receptor-mediated endocytosis
**Uptake capacity**	Abundant & promiscuous targets - low saturability	Limited by number and cycling of M6PR
**Anti-drug antibody interference**	RTB supports uptake in presence of ADA	ADA to M6P-ERT can block uptake & alter biodistribution
**Intracellular Vesicular Trafficking**		
**Endosome to lysosome pathway**	Significant: 30–70% depending on cell type	Dominant route–M6P is key lysosomal delivery signal
**Transcytosis pathway**	Significant: 30–60% depending on cell type	Minimal route
**Retrograde pathway to endoplasmic reticulum**	Minimal route (generally <5%)	Minimal route
**Zymogen Processing in Lysosomes**		
**Removal of targeting component**	RTB cleaved from enzyme and rapidly degraded	Phosphate group removed enzymatically from M6P
**Proteolytic “activation”**	Hydrolase processed to mature forms	Hydrolase processed to mature forms

## Abbreviations

AMT	Adsorptive-Mediated Transcytosis
BBB	Blood–Brain Barrier
CNS	Central Nervous System
CRIM-	Cross-Reactive Immunological Material negative
ERT	Enzyme Replacement Therapy
LSD	Lysosomal Storage Disorders
M6PR	Mannose-6-phosphate Receptors
MMR	High Mannose Receptors
MPS	Mucopolysaccharidosis
RMT	Receptor-Mediated Transcytosis
RTB	Ricin B Domain

## References

[1] Abajobir A.A., Abate K.H., Abd-Allah F., Abdulle A.M., Abera S.F., Abyu G.Y., Ahmed M.B., Aichour A.N., Aichour I., GBD 2015 Neurological Disorders Collaborator Group, V.L. (2017). Global, regional, and national burden of neurological disorders during 1990–2015: A systematic analysis for the Global Burden of Disease Study 2015. Lancet. Neurol..

[2] Abbott N.J., Patabendige A.A.K., Dolman D.E.M., Yusof S.R., Begley D.J. (2010). Structure and function of the blood–brain barrier. Neurobiol. Dis..

[3] Begley D.J., Pontikis C.C., Scarpa M. (2008). Lysosomal storage diseases and the blood-brain barrier. Curr. Pharm. Des..

[4] Pardridge W. (2006). Molecular Trojan horses for blood–brain barrier drug delivery. Curr. Opin. Pharmacol..

[5] Platt F.M., D’Azzo A., Davidson B.L., Neufeld E.F., Tifft C.J. (2018). Lysosomal storage diseases. Nat. Rev. Dis. Prim..

[6] Shachar T., Lo Bianco C., Recchia A., Wiessner C., Raas-Rothschild A., Futerman A.H. (2011). Lysosomal storage disorders and Parkinson’s disease: Gaucher disease and beyond. Mov. Disord..

[7] Desnick R.J., Astrin K.H., Schuchman E.H. (2019). Therapies for lysosomal storage diseases. Emery and Rimoin’s Principles and Practice of Medical Genetics and Genomics.

[8] Braulke T., Bonifacino J.S. (2009). Sorting of lysosomal proteins. Biochim. Biophys. Acta Mol. Cell Res..

[9] Ghosh P., Dahms N.M., Kornfeld S. (2003). Mannose 6-phosphate receptors: New twists in the tale. Nat. Rev. Mol. Cell Biol..

[10] Pryor P.R., Luzio J.P. (2009). Delivery of endocytosed membrane proteins to the lysosome. Biochim. Biophys. Acta - Mol. Cell Res..

[11] Rozaklis T., Beard H., Hassiotis S., Garcia A., Tonini M., Luck A., Pan J., Lamsa J., Hopwood J., Hemsley K. (2011). Impact of high-dose, chemically modified sulfamidase on pathology in a murine model of MPS IIIA. Exp Neurol.

[12] Schiffmann R., Hughes D.A., Linthorst G.E., Ortiz A., Svarstad E., Warnock D.G., West M.L., Wanner C., Bichet D.G., Christensen E.I. (2017). Screening, diagnosis, and management of patients with Fabry disease: Conclusions from a “kidney disease: Improving global outcomes” (KDIGO) Controversies Conference. Kidney Int..

[13] Chen J.C., Luu A.R., Wise N., De Angelis R., Agrawal V., Mangini L., Vincelette J., Handyside B., Sterling H.J., Lo M.J. (2019). Intracerebroventricular enzyme replacement therapy with Beta-Galactosidase reverses brain pathologies due to GM1 gangliosidosis in mice. J. Biol. Chem..

[14] Shen J.S., Busch A., Day T.S., Meng X.L., Yu C.I., Dabrowska-Schlepp P., Fode B., Niederkrüger H., Forni S., Chen S. (2016). Mannose receptor-mediated delivery of moss-made α-galactosidase A efficiently corrects enzyme deficiency in Fabry mice. J. Inherit. Metab. Dis..

[15] Ponder K.P. (2008). Immune response hinders therapy for lysosomal storage diseases. J. Clin. Investig..

[16] Wang J., Lozier J., Johnson G., Kirshner S., Verthelyi D., Pariser A., Shores E., Rosenberg A. (2008). Neutralizing antibodies to therapeutic enzymes: Considerations for testing, prevention and treatment. Nat. Biotechnol..

[17] Xue Y., Richards S.M., Mahmood A., Cox G.F. (2016). Effect of anti-laronidase antibodies on efficacy and safety of laronidase enzyme replacement therapy for MPS I: A comprehensive meta-analysis of pooled data from multiple studies. Mol. Genet. Metab..

[18] Nayak S., Doerfler P.A., Porvasnik S.L., Cloutier D.D., Khanna R., Valenzano K.J., Herzog R.W., Byrne B.J. (2014). Immune responses and hypercoagulation in ERT for Pompe disease are mutation and rhGAA dose dependent. PLoS ONE.

[19] Kishnani P.S., Goldenberg P.C., DeArmey S.L., Heller J., Benjamin D., Young S., Bali D., Smith S.A., Li J.S., Mandel H. (2010). Cross-reactive immunologic material status affects treatment outcomes in Pompe disease infants. Mol. Genet. Metab..

[20] Giugliani R., Alves Vieira T., Carvalho C.G., Muñoz-Rojas M.-V., Semyachkina A.N., Voinova V.Y., Richards S., Cox G.F., Xue Y. (2017). Immune tolerance induction for laronidase treatment in mucopolysaccharidosis I. Mol. Genet. Metab. Rep..

[21] Xu F., Ding E., Liao S.X., Migone F., Dai J., Schneider A., Serra D., Chen Y.T. (2004). Amalfitano, a Improved efficacy of gene therapy approaches for Pompe disease using a new, immune-deficient GSD-II mouse model. Gene Ther..

[22] Sharon N. (2007). Lectins: Carbohydrate-specific reagents and biological recognition molecules. J. Biol. Chem..

[23] Bies C., Lehr C.M., Woodley J.F. (2004). Lectin-mediated drug targeting: History and applications. Adv. Drug Deliv. Rev..

[24] Broadwell R.D., Balin B.J., Salcmant M. (1988). Transcytotic pathway for blood-borne protein through the blood-brain barrier. Proc. Natl. Acad. Sci. USA.

[25] Bell C.L., Gurda B.L., Van Vliet K., Agbandje-McKenna M., Wilson J.M. (2012). Identification of the galactose binding domain of the adeno-associated virus serotype 9 capsid. J. Virol..

[26] Wang D., Li S., Gessler D.J., Xie J., Zhong L., Li J., Tran K., Van Vliet K., Ren L., Su Q. (2018). A rationally engineered capsid variant of AAV9 for systemic CNS-directed and peripheral tissue-detargeted gene delivery in neonates. Mol. Ther. Methods Clin. Dev..

[27] Peumans W.J., Van Damme E.J. (1995). Lectins as plant defense proteins. Plant Physiol..

[28] Spooner R., Lord J. (2015). Ricin trafficking in cells. Toxins (Basel).

[29] Embleton M.J., Charleston A., Robins R.A., Pimm M.V., Baldwin R.W. (1991). Recombinant ricin toxin a chain cytotoxicity against carcinoembryonic antigen expressing tumour cells mediated by a bispecific monoclonal antibody and its potentiation by ricin toxin B chain. Br. J. Cancer.

[30] Shapira A., Benhar I. (2010). Toxin-based therapeutic approaches. Toxins (Basel).

[31] Sandvig K., Kavaliauskiene S., Skotland T. (2018). Clathrin-independent endocytosis: An increasing degree of complexity. Histochem. Cell Biol..

[32] Sandvig K., van Deurs B. (2005). Delivery into cells: Lessons learned from plant and bacterial toxins. Gene Ther..

[33] Rutenber E., Robertus J.D. (1991). Structure of ricin B-chain at 2.5 Å resolution. Proteins Struct. Funct. Bioinforma..

[34] Sphyris N., Lord J.M., Wales R., Roberts L.M. (1995). Mutational analysis of the *Ricinus* lectin B-chains. J. Biol. Chem..

[35] Sandvig K., Olsnes S., Pihl A. (1976). Kinetics of binding of the toxic lectins abrin and ricin to surface receptors of human cells. J. Biol. Chem..

[36] Lord J.M., Spooner R.A. (2011). Ricin trafficking in plant and mammalian cells. Toxins (Basel).

[37] Olsnes S. (2004). The history of ricin, abrin and related toxins. Toxicon.

[38] Sandvig K., Bergan J., Kavaliauskiene S., Skotland T. (2014). Lipid requirements for entry of protein toxins into cells. Prog. Lipid Res..

[39] Sowa-Rogozińska N., Sominka H., Nowakowska-Gołacka J., Sandvig K., Słomińska-Wojewódzka M. (2019). Intracellular transport and cytotoxicity of the protein toxin ricin. Toxins (Basel).

[40] Sandvig K., van Deurs B. (1999). Endocytosis and intracellular transport of ricin: Recent discoveries. FEBS Lett..

[41] Howes M.T., Kirkham M., Riches J., Cortese K., Walser P.J., Simpson F., Hill M.M., Jones A., Lundmark R., Lindsay M.R. (2010). Clathrin-independent carriers form a high capacity endocytic sorting system at the leading edge of migrating cells. J. Cell Biol..

[42] Mayor S., Pagano R.E. (2007). Pathways of clathrin-independent endocytosis. Mol. Cell Biol..

[43] Sandvig K., Pust S., Skotland T., van Deurs B. (2011). Clathrin-independent endocytosis: Mechanisms and function. Curr. Opin. Cell Biol..

[44] Hemalatha A., Mayor S. (2019). Recent advances in clathrin-independent endocytosis. F1000Research.

[45] Llorente A., Rapak A., Schmid S.L., van Deurs B., Sandvig K. (1998). Expression of mutant dynamin inhibits toxicity and transport of endocytosed ricin to the Golgi apparatus. J. Cell Biol..

[46] van Deurs B., Tønnessen T.I., Petersen O.W., Sandvig K., Olsnes S. (1986). Routing of internalized ricin and ricin conjugates to the Golgi complex. J. Cell Biol..

[47] Flora A.D., Teel L.D., Smith M.A., Sinclair J.F., Melton-Celsa A.R., O’Brien A.D. (2013). Ricin crosses polarized human intestinal cells and intestines of ricin-gavaged mice without evident damage and then disseminates to mouse kidneys. PLoS ONE.

[48] Audi J., Belson M., Patel M., Schier J., Osterloh J. (2005). Ricin poisoning: A comprehensive review. J. Am. Med. Assoc..

[49] Choi N.-W., Estes M.K., Langridge W.H.R. (2006). Mucosal immunization with a ricin toxin B subunit-rotavirus NSP4 fusion protein stimulates a Th1 lymphocyte response. J. Biotechnol..

[50] Medina-Bolivar F., Wright R., Funk V., Sentz D., Barroso L., Wilkins T.D., Petri W., Cramer C.L. (2003). A non-toxic lectin for antigen delivery of plant-based mucosal vaccines. Vaccine.

[51] Dorsey R., Emmett G., Salem H. (2015). Ricin. Handb. Toxicol. Chem. Warf. Agents.

[52] Boado R.J., Hui E.K.-W., Lu J.Z., Zhou Q.-H., Pardridge W.M. (2011). Reversal of lysosomal storage in brain of adult MPS-I mice with intravenous Trojan horse-Iduronidase fusion protein. Mol. Pharm..

[53] Pardridge W.M. (2017). Brain and organ uptake in the Rhesus monkey in vivo of recombinant iduronidase compared to an insulin receptor antibody-iduronidase fusion protein. Mol. Pharm..

[54] Iversen T.G., Frerker N., Sandvig K. (2012). Uptake of ricinB-quantum dot nanoparticles by a macropinocytosis-like mechanism. J. Nanobiotechnology.

[55] O’Hara J., Yermakova A., Mantis N. (2012). Immunity to ricin: Fundamental insights into toxin–antibody interactions. Curr. Top. Microbiol. Immunol..

[56] McGuinness C.R., Mantis N.J. (2006). Characterization of a novel high-affinity monoclonal immunoglobulin G Antibody against the ricin B subunit. Infect. Immun..

[57] Yermakova A., Mantis N.J. (2011). Protective immunity to ricin toxin conferred by antibodies against the toxin’s binding subunit (RTB). Vaccine.

[58] Acosta W., Ayala J., Dolan M.C., Cramer C.L. (2015). RTB Lectin: A novel receptor-independent delivery system for lysosomal enzyme replacement therapies. Sci. Rep..

[59] Condori J., Acosta W., Ayala J., Katta V., Flory A., Martin R., Radin J., Cramer C.L., Radin D.N. (2016). Enzyme replacement for GM1-gangliosidosis: Uptake, lysosomal activation, and cellular disease correction using a novel β-galactosidase:RTB lectin fusion. Mol. Genet. Metab..

[60] Condori J., Katta V., Acosta W., Ayala J., Flory A., Radin J., Davis S., Cramer C.L., Radin D.N. (2016). Novel bioproduction and delivery strategies for MPS IIIA enzyme replacement therapeutics. Mol. Genet. Metab..

[61] Martin R., Acosta W., Ayala J., Katta V., Flory A., Radin J., Devaiah S., Cramer C., Radin D. (2017). Receptor-independent mechanisms of RTB lectin-mediated ERT delivery provide unique advantages in enzyme uptake capacity, transcytosis, and lysosomal correction. Mol. Genet. Metab..

[62] Ayala J., Acosta W., Condori J., Annunziata I., Katta V., Flory A., Martin R., Radin J., Cramer C.L., d’Azzo A. (2016). Uptake, lysosomal activation, and disease correction in GM1 gangliosidosis cells by plant-made β-galactosidase: Lectin fusions. Mol. Genet. Metab..

[63] Acosta W.L., Ou L., Ayala J., Condori J., Katta V., Flory A., Martin R., Radin J., Whitley C.B., Cramer C.L. (2016). Lectin-mediated delivery of α-L-iduronidase: A novel approach for MPS I enzyme replacement therapy. Mol. Genet. Metab..

[64] Acosta W., Devaiah S., Yang T., Ayala J., Martin R., Riley K., Jarrett P., Steele J., O’Keefe K., Cramer C.L., Dickson P., Clarke L., Bigger B.W. (2018). RTB-lectin facilitates delivery of enzymes across the blood-brain-barrier in MPSI and MPSIIIA mice models. Proceedings of the 15th International Symposium on MPS and Related Diseases.

[65] Ou L., Przybilla M.J., Koniar B., Whitley C.B. (2018). RTB lectin-mediated delivery of lysosomal α-l-iduronidase mitigates disease manifestations systemically including the central nervous system. Mol. Genet. Metab..

[66] Baldo G., Mayer F.Q., Martinelli B.Z., de Carvalho T.G., Meyer F.S., de Oliveira P.G., Meurer L., Tavares Â., Matte U., Giugliani R. (2013). Enzyme replacement therapy started at birth improves outcome in difficult-to-treat organs in mucopolysaccharidosis I mice. Mol. Genet. Metab..

[67] Ou L., Herzog T., Koniar B.L., Gunther R., Whitley C.B. (2014). High-dose enzyme replacement therapy in murine Hurler syndrome. Mol. Genet. Metab..

[68] Le S.Q., Kan S., Clarke D., Sanghez V., Egeland M., Vondrak K.N., Doherty T.M., Vera M.U., Iacovino M., Cooper J.D. (2018). A humoral immune response alters the distribution of enzyme replacement therapy in murine mucopolysaccharidosis type I. Mol. Ther. - Methods Clin. Dev..

[69] Ou L., Acosta W., Koniar B.L., Cooksley R.D., Cramer C.L., Radin D.N., Whitley C.B. (2016). Enzyme replacement therapy with α-L-iduronidase and lectin RTB fusion protein in treating murine Hurler syndrome. Mol. Genet. Metab..

[70] Yermakova A., Klokk T.I., O’Hara J.M., Cole R., Sandvig K., Mantis N.J. (2016). Neutralizing monoclonal antibodies against disparate epitopes on ricin toxin’s enzymatic subunit interfere with intracellular toxin transport. Sci. Rep..

[71] Radin D., Acosta W. (2017). Materials and methods for mitigating immune-sensitization. U.S. Patent.

[72] Cohen O., Mechaly A., Sabo T., Alcalay R., Aloni-Grinstein R., Seliger N., Kronman C., Mazor O. (2014). Characterization and epitope mapping of the polyclonal antibody repertoire elicited by ricin holotoxin-based vaccination. Clin. Vaccine Immunol..

[73] Montfort W., Villafranca J.E., Monzingo A.F., Ernst S.R., Katzin B., Rutenber E., Xuong N.H., Hamlin R., Robertus J.D. (1987). The three-dimensional structure of ricin at 2.8 A. J. Biol. Chem..

[74] Grabowski G. (2008). Treatment perspectives for the lysosomal storage diseases. Expert Opin Emerg Drugs.

